# Dexmedetomidine Ameliorates Hippocampus Injury and Cognitive Dysfunction Induced by Hepatic Ischemia/Reperfusion by Activating SIRT3-Mediated Mitophagy and Inhibiting Activation of the NLRP3 Inflammasome in Young Rats

**DOI:** 10.1155/2020/7385458

**Published:** 2020-11-20

**Authors:** Wenli Yu, Jingshu Lyu, Lili Jia, Mingwei Sheng, Hongli Yu, Hongyin Du

**Affiliations:** ^1^Department of Anesthesiology, Tianjin First Center Hospital, Tianjin 300192, China; ^2^Tianjin Medical University First Center Clinical College, Tianjin 300070, China

## Abstract

Hepatic ischemia-reperfusion (HIR) has been proven to trigger oxidative stress and pyroptosis in the hippocampus. Sirtuin 3 (SIRT3) is an essential mitochondrial protein deacetylase regulating oxidative stress and mitophagy. Dexmedetomidine (Dex) has been demonstrated to confer neuroprotection in different brain injury models. However, whether the protective effects of Dex following HIR are orchestrated by activation of SIRT3-mediated mitophagy and inhibition of NOD-like receptor protein 3 (NLRP3) inflammasome activation remains unknown. Herein, two-week-old rats were treated with Dex or a selective SIRT3 inhibitor (3-TYP)/autophagy inhibitor (3-MA) and then subjected to HIR. The results revealed that Dex treatment effectively attenuated neuroinflammation and cognitive deficits via upregulating SIRT3 expression and activity. Furthermore, Dex treatment inhibited the activation of NLRP3 inflammasome, while 3-TYP and 3-MA eliminated the protective effects of Dex, suggesting that SIRT3-mediated mitophagy executes the protective effects of Dex. Moreover, 3-TYP treatment downregulated the expression level of SIRT3 downstream proteins: forkhead-box-protein 3*α* (FOXO3*α*), superoxide dismutase 2 (SOD2), peroxiredoxin 3 (PRDX3), and cyclophilin D (CYP-D), which were barely influenced by 3-MA treatment. Notably, both 3-TYP and 3-MA were able to offset the antioxidative and antiapoptosis effects of Dex, indicating that SIRT3-mediated mitophagy may be the last step and the major pathway executing the neuroprotective effects of Dex. In conclusion, Dex inhibits HIR-induced NLRP3 inflammasome activation mainly by triggering SIRT3-mediated mitophagy.

## 1. Introduction

Liver transplantation is the standard treatment for children with advanced liver disease. Although the 1- and 5-year survival rates after liver transplantation are currently at about 95% and 85%, respectively [1], neurological complications are still prevalent after surgery and affect 15%-30% of liver transplant recipients [2]. Additionally, pediatric patients tend to be more sensitive and are prone to higher mortality rates compared to adults [3, 4]. What is more, neurological injuries in early life translate to long-lasting developmental consequences [5]. Hepatic ischemia-reperfusion (HIR) is an essential step in liver transplantation and has an important effect on early liver allograft dysfunction and long-term graft survival. This not only mediates liver but also remote organ injury [6, 7]. Previous studies by our research group have revealed that this process is able to cause damage to the hippocampus and results in long-time cognitive function in young mice [8–10]. However, the mechanisms involved in hippocampus injury induced by HIR remain unclear.

Mitochondria are considered as unique and irreplaceable organelles in eukaryotic cells. They play vital roles in the generation of energy and reactive oxygen species (ROS) [11]. Previous research found that mitochondrial dysfunction and pyroptosis were involved in hippocampus injury induced by HIR [9, 12]. The damaged mitochondria were reported to cause an imbalance in ROS homeostasis and subsequent accumulation of ROS. Additionally, previous reports demonstrated that mitochondrial dysfunction could activate the NLRP3 inflammasome by generating ROS [13, 14]. However, mitophagy negatively regulated inflammasome activity by scavenging ROS in the mitochondria [15].

The sirtuin family comprises seven members, SIRT1-7, among which sirtuin 1 (SIRT1) is most extensively studied because of its diverse roles in mitochondrial biogenesis and function, cell metabolism, and inflammation [16–18]. A lot of research has been done to prove SIRT1 in protecting the brain [19], heart [20], liver [21], and kidney [22] against ischemia-reperfusion injury. However, the role of sirtuin 2 (SIRT2), the most abundant sirtuin protein in the brain, is still controversial [23]. The inhibition of SIRT2 alleviates neuroinflammation induced by lipopolysaccharide [24]. In contrast, SIRT2 has recently been shown to be an acetylation switch of the NLRP3 inflammasome to repress its activation [25, 26]. SIRT3 is a key mitochondrial protein deacetylase, highly expressed in the brain [27], which has significant effects on oxidative stress, mitochondrial function, mitophagy, pyroptosis, and apoptosis [28]. Recent research proved that increasing SIRT3 levels alleviated mitochondrial dysfunction by normalizing mitochondrial bioenergetics in Parkinson's [29] and Alzheimer's diseases [30]. Moreover, overexpression of SIRT3 was shown to protect multiple organs from prolonged ischemia-reperfusion injury [31–34]. However, whether SIRT3-mediated mitophagy plays a part in hippocampus injury induced by HIR remains largely unclear.

Dexmedetomidine (Dex) is a type of general anesthetic, widely used in children and infants [35, 36]. Numerous studies have elucidated its anti-inflammatory, antiapoptotic, and neuroprotective effects [37]. Recently, Dex was reported to inhibit the activation of the NLRP3 inflammasome and thereby inhibiting brain injury [38, 39]. Additionally, the most recent report published in the British Journal of Anaesthesia showed that Dex could upregulate SIRT3 and in turn inhibiting mitochondrial damage and cell apoptosis in renal ischemia-reperfusion injury [40]. Based on the aforementioned evidence, this study hypothesized that Dex could exert neuroprotective effects by upregulating SIRT3-mediated mitophagy and inhibiting activation of the NLRP3 inflammasome. Therefore, the present study used two-week-old rat models to explore the influence of Dex on SIRT3. Additionally, the relationship between Dex and the NLRP3 inflammasome with regard to hippocampus injury and cognitive dysfunction induced by HIR was investigated.

## 2. Materials and Methods

### 2.1. Animals

Sprague-Dawley rats (aged 2 weeks, weighing around 20 g) were purchased from the Beijing HFK Bioscience Company (Beijing, China). They were housed in a temperature- and humidity-controlled environment and fed *ad libitum*. All animal procedures were approved by the Ethics Committee of Tianjin First Center Hospital (Tianjin, China) and complied with the National Institute of Health's Guidelines for the Care and Use of Laboratory Animals.

### 2.2. Animal Groups

To explore the time-dependent effects of HIR on sirtuin expression, the rats were randomly allocated into two groups: sham operation group (S group) (*n* = 10) and hepatic ischemia-reperfusion group (IR group). The IR group was further divided into five subgroups (*n* = 10/subgroup) according to sample collection time points (2 h, 6 h, 1 d, 3 d, and 7 d) after reperfusion. Additionally, the selective SIRT3 inhibitor 3-TYP (Selleck, Shanghai, China) and autophagy inhibitor 3-MA (Selleck, Shanghai, China) were pretreated for further experiments. 3-TYP (50 mg/kg), 3-MA (2 mg/kg), or vehicle (1% ethanol) of equal volume were intraperitoneally (i.p.) injected at a dose of every two days for a total of three doses before modeling [41]. Moreover, the rats received an intraperitoneal injection of 25 *μ*g/kg Dex (Hengrui Pharmaceutical Co., Ltd., Jiangsu, China) which was dissolved in normal saline at a fixed proportion of body weight (100 *μ*L/10 g) 30 min before the operation [42]. Notably, during preliminary studies, it was shown that neither the inhibitor nor the vehicle resulted to significant adverse effects on the sham-operated rats (as shown in Supplementary Information). Therefore, the rats were further randomly divided into the following groups (*n* = 50): (1) S group, (2) IR group, (3) DEX group where they were treated with 25 *μ*g/kg Dex before hepatic ischemia, (4) 3-TYP group where they were pretreated with 3-TYP then treated with 20 *μ*g/kg Dex before hepatic ischemia, and (5) 3-MA group where rats were pretreated with 3-MA then treated with 25 *μ*g/kg Dex before hepatic ischemia. Finally, blood and tissue samples were harvested from rats 3 d after reperfusion.

### 2.3. Construction of the 70% Warm HIR Model

The rats were subjected to HIR as previously described [9]. Briefly, they were subjected to a fast the night before the experimental operation. The rats were however allowed to take water. Afterwards, all the animals were deeply anesthetized with amyl sodium pentobarbital (1% and 30mg/kg, i.p.). A heating lamp was then used to provide sufficient warmth in order to relieve the animals of stress from the anesthetic. Following this, the ventral midline was incised for entry into the abdominal cavity to expose the liver. All structures in the portal triad (hepatic artery, portal vein, and bile duct) to the left and median liver lobes were then occluded with a microvascular clamp to establish 70% hepatic ischemia. Successful occlusion was confirmed by the appearance of the pallor of the left and median liver lobes. Afterwards, the abdominal incision was covered with a wet and heated sterilized gauze to minimize fluid and heat loss. Moreover, hepatic reperfusion was initiated by removing the clamp after 60min of ischemia; then, the abdominal cavity was flushed with a sterilized gauze and closed using a sterilized surgical silk. Rats in the sham operation group only underwent separating vessels and bile duct pedicles without clamping of the blood vessels. Additionally, most rats were sacrificed three days after reperfusion, except for those in the first experiment. Blood samples were obtained from the inferior vena cava after anaesthesia, and serum was extracted by centrifugation at 3000×g for 15min. Furthermore, brain tissues were collected from rats after transcardial perfusion with chilled phosphate-buffered saline (50mM PBS and pH=7.4); then, hippocampal tissues were isolated.

### 2.4. ELISA

The serological markers for brain injury, namely, S100*β* and neuron-specific enolase (NSE) as well as pyroptosis indicators including interleukin-1*β* (IL-1*β*) and interleukin-18 (IL-18), were detected in the hippocampus using the corresponding enzyme-linked immunosorbent assay (ELISA) kit (Shanghai Biovol Corp., Shanghai, China) following the manufacturer instructions.

### 2.5. Measurement of SIRT1 and SIRT3 Activity

Hippocampal mitochondria were isolated using the tissue mitochondria isolation kit (Sigma, USA). Afterwards, the bicinchoninic acid (BCA) protein assay was performed to determine protein concentration. SIRT1 and SIRT3 activities were then measured using the SIRT1 and SIRT3 Fluorimetric Activity Assay Kit (Abcam, USA) according to the manufacturer's protocol. Briefly, the mitochondrial extract was incubated with a mixture of SIRT1 or SIRT3 assay buffer, fluoro-substrate peptide, NAD, and developer at room temperature for 1 h. Fluorescent intensity was then measured at 485 nm/535 nm (SIRT1) or 350 nm/450 nm(SIRT3) using a microtiter plate fluorometer (23225, Thermo Fisher Scientific, Waltham, MA, USA).

### 2.6. Determination of Oxidative Indexes

Oxidative indexes in the hippocampus including superoxide dismutase (SOD), ROS, and malondialdehyde (MDA) were detected using the corresponding detection kits based on the manufacturer's recommendations (Nanjing Jiancheng Bioengineering Institute, Nanjing, China).

### 2.7. Measurement of Mitochondrial Function

Isolated hippocampal mitochondria were used to determine mitochondrial function. This entailed an analysis of ATP levels, mitochondrial membrane potential (MMP), and morphological changes in the mitochondria.

ATP levels were measured using a firefly luciferase-based ATP assay kit (Beyotime, China). Firefly luciferase catalyzes the production of fluorescence by fluorescein, and this requires energy from ATP. Therefore, when both luciferase and luciferin are in excess, generation of fluorescence is proportional to the concentration of ATP. Consequently, ATP levels in the sample could be calculated according to the standard curve which was based on changes in the intensity of fluorescence.

Additionally, MMP was evaluated using an MMP assay kit with JC-1 (Beyotime, China). Briefly, JC-1 gathered in the mitochondrial matrix to form a polymer (red fluorescence, excitation at 525 nm and emission at 590 nm) when the MMP was high. However, when the situation was reversed, JC-1 was internalized as a monomer (green fluorescence, excitation at 490 nm and emission at 530 nm). The ratio of green to red fluorescence was then calculated, indicating the loss of MMP.

Furthermore, transmission electron microscopy (TEM) was utilized to observe morphology of the mitochondria and the extent of autophagy/mitophagy. Briefly, the middle segments of the hippocampus were cut into small tissue blocks (≈1 mm^3^), followed by an immersion in 2% cacodylate-buffered glutaraldehyde for 6 h. They were then rinsed three times with PBS and postfixed for 2 h with 1% osmium tetraoxide. After fixation, the tissues were dehydrated in a graded series of alcohol and embedded in Epon. Finally, samples were poststained with lead citrate and uranyl acetate. Morphology of the mitochondria was then observed under a TEM (Hitachi HT7700, Japan).

### 2.8. Immunoprecipitation

Hippocampal tissues were immersed in lysis buffer and crushed ultrasonically. They were then lysed on ice for 30 mins with continuous shaking to ensure the cells were fully disintegrated. After lysis, the mixture was centrifuged at 12,000 rpm for 15 min to obtain the supernatants. The supernatants were then incubated with the CYP-D antibody overnight (1 : 800, CST, USA) at 4°C, while slowly shaking, followed by an incubation with protein A/G agarose beads (Thermo, Lot: PA195905) at 4°C for 24 h. After the immune precipitation reaction, the beads were rinsed three times in RIPA buffer, resuspended in Laemmli buffer, and boiled for subsequent Western blot (WB) analysis. The primary antibodies used were CYP-D (1 : 800, CST, USA) and the acetylated-lysine antibody (1 : 800, CST, USA).

### 2.9. Immunofluorescence

Brain tissue paraffin slides were deparaffinized, rehydrated, antigen retrieved, and incubated with 3% hydrogen peroxide and 10% normal goat serum, similar to procedures performed in the immunohistochemistry assay. The sections were incubated with primary antibodies against Iba-1 (1 : 200, Abcam, USA) at 4°C overnight and rinsed with PBST 3 times. The following procedures were performed in darkness. The slides were incubated with corresponding secondary antibody Alexa Fluor 488-conjugated anti-rabbit IgG+Alexa CY3-conjugated anti-mice IgG at 37°C for 30 minutes and then rinsed. Finally, the slides were counterstained with DAPI for 10 min to visualize nuclear, and images were captured under a fluorescence microscope (Olympus, Tokyo, Japan).

### 2.10. Immunohistochemistry

Brain tissue paraffin slides were heated at 65°C for 40 min, deparaffinized, and then rehydrated. The slides were immersed in citrate buffer (pH 6.0) and heated at 99°C for 18 min in a microwave oven for antigen retrieval, then cooled at room temperature. Endogenous peroxidase activity was quenched by treatment with 3% hydrogen peroxide (Zsbio, China) for 15 min at room temperature, and nonspecific binding sites were blocked by treatment with 10% normal goat serum (Zsbio, China) for 30 min at 37°C. Slides were incubated with primary antibody against NLRP3 (1 : 200, Abcam, USA) overnight at 4°C. The slides were subsequently incubated with biotin-labeled secondary antibody and streptavidin working solution (Zsbio, China) for 15 min. Next, the slides were visualized with 3,3′-diaminobenzidine (DAB) substrate chromogen system (Zsbio, China), rinsed with tap water, and counterstained with hematoxylin. Finally, immunoreactive signals were observed under a light microscope (Olympus, Tokyo, Japan).

### 2.11. TUNEL Staining

Neuronal apoptosis was assessed by the TUNEL (terminal digoxigenin-labeled dUTP nick-end labeling) assay, which was performed using ApopTag Plus peroxidase In Situ Apoptosis Detection kit (Roche, Swiss Basel) according to the manufacturer's instructions. Samples were observed under a fluorescence microscope (red fluorescence, excitation at 450~500 nm and detection at 515~565 nm; Olympus, Japan) after a labeling reaction step.

### 2.12. Western Blot

Proteins were extracted from hippocampal tissues with RIPA lysis buffer (Solarbio, China) containing the protein phosphatase inhibitor complex (Biomed, China) and phenylmethylsulfonyl fluoride (PMSF, BestBio, China). The concentration of the protein extracts was determined using the BCA protein assay (23225, Thermo Fisher Scientific, Waltham, MA, USA). The proteins were separated by sodium dodecyl sulfate-polyacrylamide gel electrophoresis (SDS-PAGE) gel (Solarbio, China) and blotted onto polyvinylidene fluoride (PVDF) membranes (Millipore, USA). After blocking with 5% nonfat milk (1X PBS and 5% nonfat dry milk) for 1 h at room temperature, the membranes were incubated with primary antibodies against the following proteins: SIRT1-3 (1 : 1000, CST, USA), SIRT6-7 (1 : 1000, CST, USA), SOD2 (1 : 1000, CST, USA), PRDX3 (1 : 1000, CST, USA), CYP-D (1 : 1000, CST, USA), FOXO3*α* (1 : 1000, CST, USA), BINP3 (1 : 1000, CST, USA), microtubule-associated protein light chain 3 (LC3) LC3-II/LC3-I (1 : 1000, CST, USA), P62 (1 : 1000, CST, USA), NLRP3 (1: 1000, Abcam, USA), cleaved-caspase-1 (1: 500, Wanleibio, China), ASC (1: 1000, Abcam, USA), and GSDMD (1: 1000, Abcam, USA) and then incubated with the appropriate secondary antibodies for 1 h at room temperature. The blots were detected by enhanced chemiluminescence (ECL) substrate (Millipore, USA). The protein bands were visualized by Molecular Imager Gel Doc XR System (Bio-Rad, Hertfordshire, UK) and quantified by densitometric analysis using an image analyzer (NIH Image J software, USA).

### 2.13. Novel Object Recognition

The novel object recognition (NOR) procedure was performed 4 weeks after HIR. It was completed in 3 days in three stages: adaptation phase, training familiarization phase, and testing phase. In the adaptation phase, each rat was placed in a 50 cm × 50 cm × 50 cm uncovered test chamber for 10 min as an introduction to the environment. During the training familiarization phase, rats were allowed to freely explore the test chamber which had two identical objects for 10 min. In the testing phase, one of the objects was replaced with an object with a different color and shape after a 1 h interval. These steps were repeated in the second day. Exploring time is defined by the amount of time the rat spent pointing the nose at an object at a distance of less than 2 cm or contacting it with the nose. The exploring time that rat spent in familiar objects and novel objects were automatically recorded by a camera system, and recognition index (RI) was calculated (RI = time exploring novel object/(time exploring novel object + time exploring familiar object) × 100%).

### 2.14. Statistics

Statistical analysis was performed by the SPSS 22.0 statistical software. Variables were described as mean ± standard deviation (SD). Mean values between groups were compared with the Student *t*-test or one-way ANOVA. *P* < 0.05 was considered statistically significant.

## 3. Results

### 3.1. HIR Induced an Increase in Serum Brain Damage Markers

**Figure 1:**
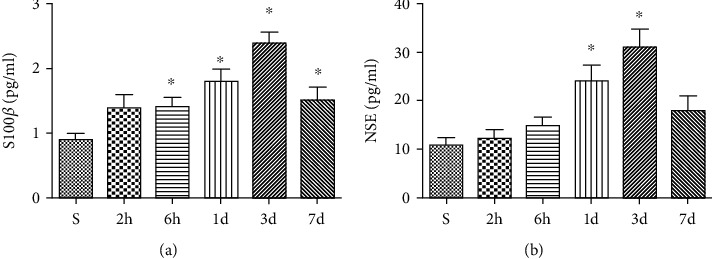
HIR induced an increase of serum brain damage markers. The levels of serum brain damage markers: (a) S100*β* and (b) NSE. *n* = 5 per group. Data are presented as mean ± SEM. ^∗^*P* < 0.05 vs. the S group.

To assess the degree of brain damage, the level of serum brain damage markers (S100*β* and NSE) was assessed. Notably, concentrations of serum S100*β* and NSE began to elevate slightly 6 h after reperfusion and reached the maximum level 3 d after reperfusion. However, the levels returned to normal 7 d after reperfusion, as shown in Figures 1(a) and 1(b).

### 3.2. Effects of HIR on Sirtuin Expression in the Hippocampus

**Figure 2:**
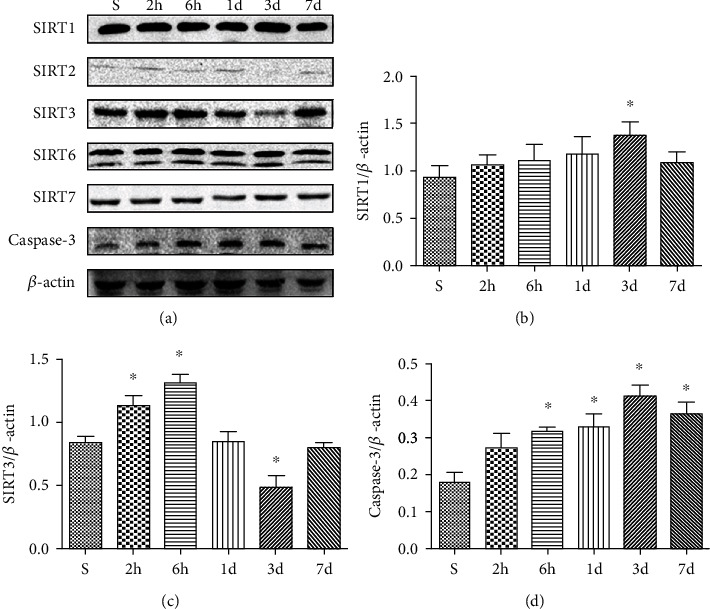
Effects of HIR on sirtuin expression. (a) Representative blots of sirtuin and caspase-3 in hippocampus tissues. (b) Quantitative analysis of the levels of SIRT1 expression. (c) Quantitative analysis of the levels of SIRT3 expression. (d) Quantitative analysis of the levels of caspase-3 expression. *n* = 5 per group. Data are presented as mean ± SEM. ^∗^*P* < 0.05 vs. the S group.

To determine whether sirtuins play a role in the hippocampus injury induced by HIR, we first determined the protein levels of SIRT1, SIRT2, SIRT3, SIRT6, and SIRT7 in the hippocampus after 60 minutes of ischemia with various lengths of reperfusion. Among the three mitochondrial sirtuins, namely, SIRT3, SIRT4, and SIRT5, previous research showed that SIRT3 regulated the majority of mitochondrial lysine acetylation [43]. So we only analyze the SIRT3 protein instead of the three. Additionally, caspase-3, a key mediator of apoptosis, was also measured to assess the apoptosis. Upon reperfusion, the SIRT1 increased slightly at 3 d after reperfusion, whereas the expression of the SIRT3 protein initially increased but gradually declined 1 d after reperfusion and reached the lowest level 3 d after reperfusion as shown in Figures 2(a)–2(c). The results showed that the expression of SIRT2 was too low to be detected. SIRT6 and SIRT7 were unaffected by HIR. However, expression of the caspase-3 protein gradually increased and reached the maximum value 3 d after reperfusion, indicating an increased level of apoptosis (Figure 2(d)). Based on the above results, it was speculated that brain damage was most acute 3 d after reperfusion because the lowest levels of the SIRT3 protein were observed at this time. Therefore, rats at 3 d after reperfusion were selected for subsequent experiments.

### 3.3. Dex Lowered Serum Brain Damage Markers and Upregulated SIRT3 Expression and Activity in the Hippocampus after HIR

**Figure 3:**
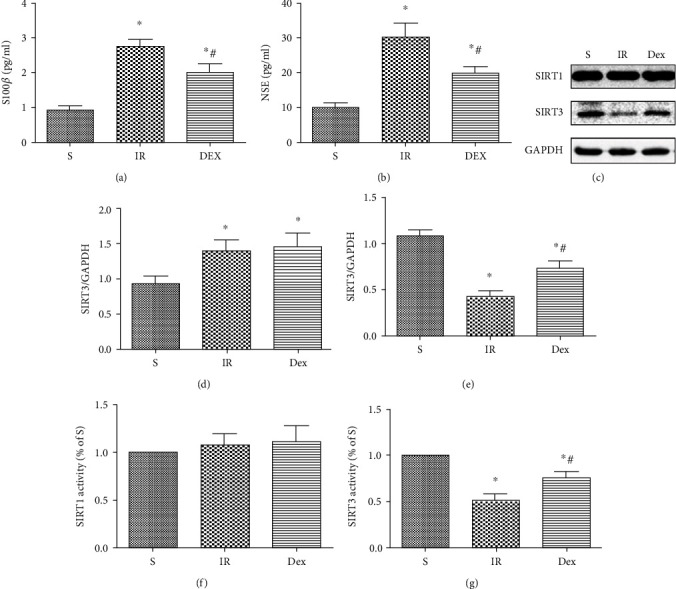
Dex decreased serum brain damage markers and upregulated SIRT3 expression and activity in the hippocampus after HIR. Serum levels of brain damage markers: (a) S100*β* and (b) NSE (b). (c) Expression of SIRT1 and SIRT3 in hippocampus tissues. (d) Quantitative analysis of expression level of SIRT1. (e) Quantitative analysis of expression level of SIRT3. (f) SIRT1 activity in mitochondria from hippocampus tissues. (g) SIRT3 activity in mitochondria from hippocampus tissues. *n* = 5 per group. Data are presented as mean ± SEM.^∗^*P* < 0.05 vs. S group; ^#^*P* < 0.05 vs. IR group.

The effect of Dex on serum brain damage markers (S100*β* and NSE) as well as the activity and expression of SIRT1 and SIRT3 3 d after reperfusion was tested. The study found that Dex treatment effectively lowered both serum S100*β* and NSE concentrations as shown in Figures 3(a) and 3(b). Additionally, as shown in Figures 3(c)–3(g), the Dex treated group showed a significant upregulation of SIRT3 expression and activity compared to the IR group. However, Dex treatment had no such effects on the SIRT1 expression and activity. To demonstrate the role of SIRT3 in the neuroprotective effects of Dex, 3-TYP, the specific inhibitor of SIRT3, was used to lower expression of the protein in subsequent experiments.

### 3.4. Activation of SIRT3 by Dex Treatment Was Involved in Improved Cognition

**Figure 4:**
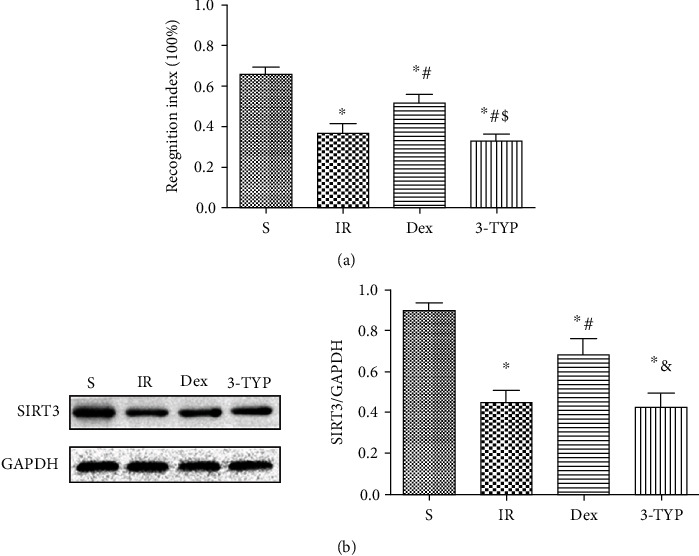
Dex improved cognition via SIRT3 activation in the hippocampus after HIR. (a) NOR test results of rats 4 weeks after HIR. (b) Representative Western blots and quantification of SIRT3 in the hippocampus 4 weeks after HIR in rats. *n* = 5 per group. Data are presented as mean ± SEM. ^∗^*P* < 0.05 vs. S group; ^#^*P* < 0.05 vs. IR group; ^&^*P* < 0 : 05 vs. Dex group.

A previous study by our research group indicated that HIR mainly interfered with the memory recall aspect but not learning ability. Therefore, the NOR test was applied to assess recognition memory 4 weeks after HIR. The study analyzed the functional relevance of the findings using NOR 4 weeks after modeling. Afterwards, the rats were sacrificed for the analysis of SIRT3 expression levels in the hippocampus using the Western blot technique. This analysis confirmed that HIR significantly lowered recognition index (RI) as well as SIRT3 expression levels. In contrast, treatment with Dex increased RI as well as the expression levels of SIRT3. However, this effect was remarkably reversed upon treatment with 3-TYP as shown in Figures 4(a) and 4(b).

### 3.5. Dex *Enhanced Mitophagy in the Hippocampus after HIR*

**Figure 5:**
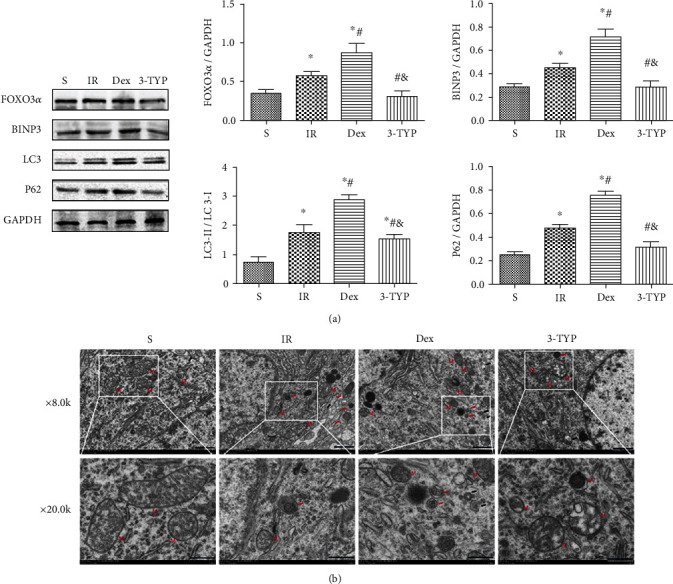
Dex enhanced mitophagy in the hippocampus after HIR. (a) The representative Western blots and quantification of mitophagy-related proteins: FOXO3*α*, BINP3, LC3-II/LC3-I, and P62. (b) The TEM images showing mitophagy in the hippocampus CA1 region (magnification ×8000 and 200000; scale bars 1 *μ*m and 500 nm). “M” represented mitochondria. Red arrows indicated mitophagy vacuoles. Black arrows indicated autophagic vacuoles. *n* = 5 per group. Data are presented as mean ± SEM. ^∗^*P* < 0.05 vs. S group; ^#^*P* < 0 : 05 vs. IR group; ^&^*P* < 0.05 vs. Dex group.

To elucidate the effect of Dex on mitophagy in the hippocampus, markers of mitophagy were analyzed through Western blot then the ultrastructure of hippocampus neurons viewed using TEM. The results revealed that HIR induced a slight increase in the expression levels of FOXO3*α*, BINP3, LC3-II/LC3-I, and P62 as shown in Figure 5(a). In contrast, treatment with Dex induced a significant increase in the mitophagy-related proteins. Additionally, as shown in Figure 5(b), TEM revealed that the number of mitophagosomes increased in the Dex group, indicating that the anesthetic enhanced mitophagy. Moreover, treatment with 3-TYP reversed the induction of mitophagy by decreasing the expression levels of FOXO3*α*, BINP3, LC3-II/LC3-I, and P62. Results from TEM further corroborated with the above findings. To further explore the relationship between Dex, SIRT3, and mitophagy, the study used 3-MA to inhibit mitophagy.

### 3.6. Dex Attenuated Neuronal Apoptosis in the Hippocampus after HIR

**Figure 6:**
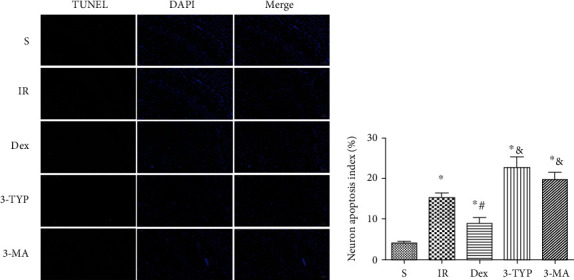
Dex attenuated neuronal apoptosis in hippocampus after HIR. Representative images show TUNEL staining in the hippocampal CA1. The number of apoptotic cells was detected by TUNEL (red pixels), and the nuclei were identified by DAPI staining (blue pixels) (magnification ×200; scale bars 50 *μ*m). Apoptosis index was calculated as follows: the number of apoptotic cells/the number of total cells × 100%.

To investigate the possible mechanisms underlying cognitive dysfunction, the study detected neuron apoptosis in hippocampal CA1 through TUNEL staining (Figure 6). The results showed that Dex treatment attenuated neuronal apoptosis in the hippocampus after HIR. Notably, treatment with 3-TYP and 3-MA largely eliminated the antiapoptotic effects of Dex.

### 3.7. Dex Inhibited Microglia Activation and Oxidative Stress in the Hippocampus after HIR

**Figure 7:**
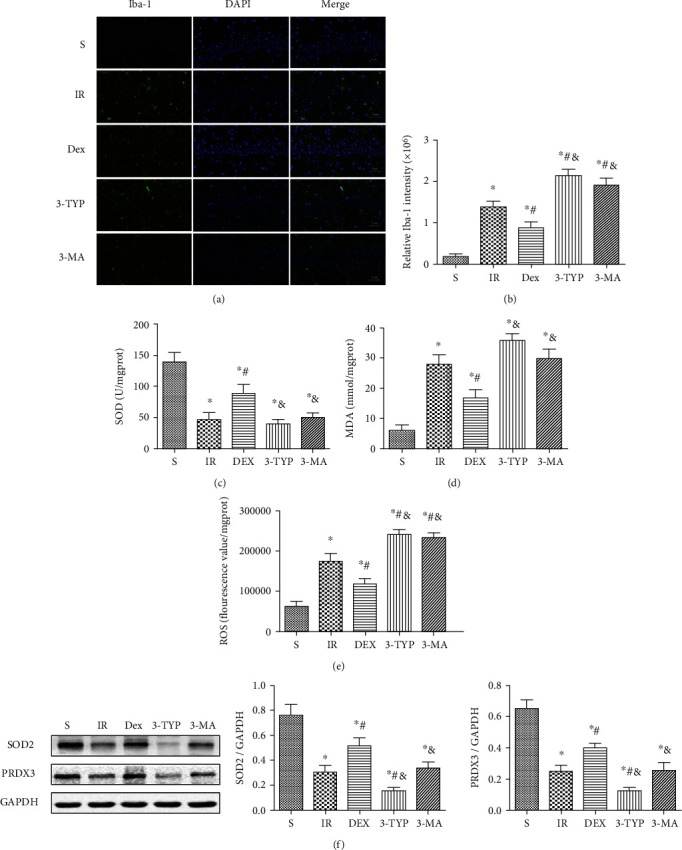
Dex inhibited microglia activation and oxidative stress in the hippocampus after HIR. (a) Representative immunofluorescence images of Iba-1 (green pixels) in the hippocampal CA1 region (magnification ×400; scale bars 50 *μ*m). (b) Quantitative analyses of the fluorescence intensity of Iba-1. The oxidative stress indexes: (c) SOD, (d) MDA, (e) and ROS in the hippocampus after HIR. (f) The representative Western blot and quantification of the main mitochondrial antioxidant enzymes: SOD2 and PRDX3. *n* = 5 per group. Data are presented as mean ± SEM. ^∗^*P* < 0.05 vs. the S group; ^#^*P* < 0.05 vs. IR group; ^&^*P* < 0.05 vs. Dex group.

Activation of the microglia is a major indicator of neuroinflammation. Consequently, the study used the specific microglial marker, Iba-1, to assess the extent of microglia activation through immunofluorescence. Additionally, ROS, SOD, and MDA were measured to evaluate the level of oxidative stress in the hippocampus after HIR. Compared to the S group, HIR dramatically enhanced the intensity of Iba-1 fluorescence in the hippocampal CA1 region, suggesting that microglia were activated after the procedure as shown in Figures 7(a) and 7(b). Moreover, levels of ROS and MDA were significantly increased with the decline of SOD activity (Figures 7(c)–7(e)). A reduction in the intensity of Iba-1 of fluorescence was also observed in the Dex-treated group, indicating that treatment with the anesthetic could inhibit the activation of microglia. Furthermore, treatment with Dex upregulated SOD activity but decreased the levels of ROS and MDA. However, treatment with 3-TYP and 3-MA prior to Dex injection eliminated the effects of the anesthetic, further enhancing microglia activation and oxidative stress.

SOD2 and PRDX3 are the major mitochondrial antioxidant enzymes that scavenge ROS and H_2_O_2_, respectively. Both can be deacetylated by SIRT3. The Western blot (Figure 7(f)) results showed that HIR resulted in the decline of SOD2 and PRDX3 while Dex treatment induced a significant upregulation of both enzymes. Notably, treatment with 3-TYP decreased the expression of both SOD2 and PRDX3 compared to the HIR group. However, 3-MA had no influence on their expression.

### 3.8. Dex Maintained Mitochondrial Function in the Hippocampus after HIR

**Figure 8:**
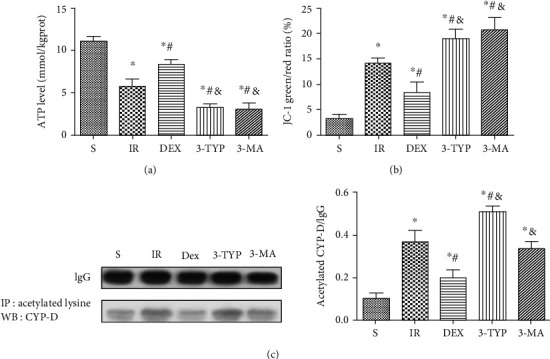
Dex maintained mitochondrial function in the hippocampus after HIR. (a) ATP content in the hippocampus. (b) Loss of MMP in the hippocampus. (c) The representative Western blot and densitometry of the CYP-D acetylation level. *n* = 5 per group. Data are presented as mean ± SEM. ^∗^*P* < 0.05 vs. S group; ^#^*P* < 0.05 vs. IR group; ^&^*P* < 0.05 vs. Dex group.

To evaluate the hippocampus mitochondrial function, levels of ATP and MMP were measured. Dex increased ATP production and restored MMP compared to the HIR group as shown in Figures 8(a) and 8(b). However, 3-TYP and 3-MA exerted the opposite effect. CYP-D is located in the mitochondrial matrix, and its hyperacetylation can trigger mPTP and collapse MMP as well as disrupt ATP production. Therefore, the study further assessed the level of CYP-D acetylation through immunoprecipitation and Western blot. Notably, HIR increased acetylation of CYP-D in the hippocampus as shown in Figure 8(c). Treatment with Dex however had an inverse effect. Moreover, 3-TYP treatment restored the high acetylation of CYP-D.

### 3.9. Dex Inhibited Activation of the NLRP3 Inflammasome in the Hippocampus after HIR

**Figure 9:**
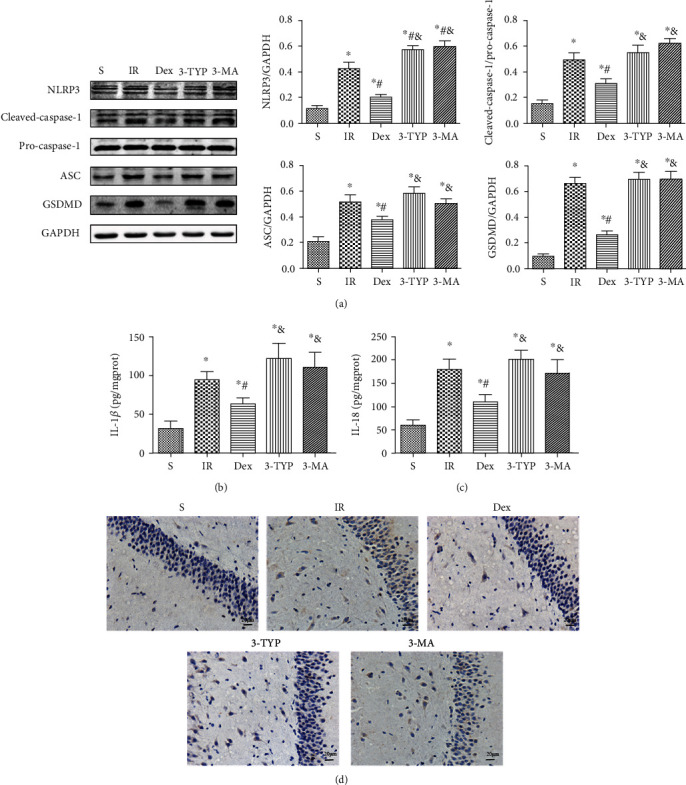
Dex inhibited activation of the NLRP3 inflammasome in the hippocampus after HIR. (a) The representative Western blot and quantification of pyroptosis-related proteins: NLRP3, cleaved-caspase-1, pro-caspase-1, ASC, and GSDMD. (b, c) Secretion levels of IL-1*β* and IL-18 in the hippocampus. (d) Representative images of immunohistochemical staining with the NLRP3 antibody in the hippocampal CA1 region (magnification ×400; scale bars 20 *μ*m). *n* = 5 per group. Data is presented as mean ± SEM. ^∗^*P* < 0.05 vs. the S group; ^#^*P* < 0.05 vs. the IR group; ^&^*P* < 0.05 vs. the Dex group.

Mitochondrial dysfunction and defects in mitophagy can induce activation of the NLRP3 inflammasome. Therefore, the study further explored whether Dex could also inhibit activation of the NLRP3 inflammasome in the hippocampus after HIR. Treatment with Dex decreased the expression levels of NLRP3, cleaved-caspase-1, pro-caspase-1, ASC, and GSDMD (Figure 9(a)). This was consistent with results of the NLRP3 immunohistochemical staining (Figure 9(d)) and decreased secretion of IL-1*β* and IL-18 in the hippocampus (Figures 9(b)–9(c)). Notably, treatment with 3-TYP and 3-MA largely reversed the inhibitory effect of Dex.

## 4. Discussion

In the recent years, HIR has been the focus of several studies due to its role in various clinical scenarios. With the implementation of vascular operation techniques in hepatic surgery, HIR is considered critical in postoperative morbidity and mortality [6]. This is because it not only causes liver dysfunction but also damages to distant organs [44, 45] especially the brain [12]. Previous studies by our research group proved that HIR induced overactivation of the N-methyl-D-aspartate (NMDA) receptor subunit 2A (NR2A), resulting to hippocampal neuron apoptosis with long-term cognitive dysfunction. This mainly damaged the memory recall aspect but had no effect on learning abilities [8]. Further research on the potential mechanisms of hippocampus injury and cognitive dysfunction induced by HIR revealed that abnormal mitochondrial dynamics may be involved in this process [9]. Additionally, activation of the NLRP3 inflammasome was proven to drive inflammation in the hippocampus and cortex [12]. These findings suggested a possible connection between the mitochondria and activation of the NLRP3 inflammasome.

The SIRT family is a group of histone deacetylases whose activities are dependent on and regulated by nicotinamide adenine dinucleotide (NAD+). They are named SIRT1-7 and have different tissue distribution, subcellular localization, enzyme activity, and target proteins [46]. The most widely studied member of the mammalian sirtuin family is SIRT1, which plays an important role in mitochondrial biogenesis [16]. Besides, SIRT2 has recently been shown to be a direct regulator of the NLRP3 inflammasome [25, 26]. Although three mitochondrial sirtuins exist, namely, SIRT3, SIRT4, and SIRT5, previous research showed that SIRT3 regulated the majority of mitochondrial lysine acetylation [43]. Moreover, recent studies reported that expression of SIRT3 was upregulated in hippocampal neurons in response to exercise. This presented a promising means of protecting the mitochondria and neurons against metabolic and excitotoxic stress [47].

In this study, the expression of SIRT2 protein in the hippocampus was too low to be detected, this finding is consistent with SIRT protein expression patterns during rat hippocampal development [48]. However, the expression of SIRT1 protein in the hippocampus increased slightly at 3 d after reperfusion, which was opposite to some previous studies [49]. The expression of the SIRT3 protein in the hippocampus initially increased as a protective response to HIR challenge. The observation was consistent with previous reports where expression of the protein was upregulated during caloric restriction [50] and intermittent fasting [51]. This enhanced mitochondrial function by promoting energy production and maintaining metabolic homeostasis [52]. However, expression of the SIRT3 protein gradually reduced from 1 d after reperfusion, reaching the lowest level at 3 d following reperfusion. Additionally, caspase-3 and the concentrations of serum brain injury markers including S100*β* and NSE peaked 3 days after reperfusion. In other ischemia-reperfusion injury models, SIRT3 was found at considerably low levels and this was closely linked to increased mitochondrial damage and organ dysfunction [14, 53, 54]. Contrary to this study, these previous reports did not observe the variation of SIRT3 expression as a function of time. These findings suggested an association among injury of the hippocampus, increased SIRT1, and reduced SIRT3 expression. Combined with the characteristics of SIRT1 expression in developing rats, we made the following hypothesis: when injury occurred, both SIRT1 and SIRT3 increased as a protective response. However, excessive damage resulted in too much consumption of SIRT3 ultimately leading to hippocampus injury. Since SIRT1 was expressed at a much higher level in developing mice than in adult mice, it will not be excessively consumed.

In addition, SIRT3 was reported to have different levels of expression in developing and adult rat brains. In the developing brain, there was a significant increase in expression of the SIRT3 protein in the cortex and hippocampus between postnatal day 7 and day 21. These levels were maintained in adulthood and old age [48]. A separate study also showed that upregulating SIRT3 in hippocampus neurons could correct deficits in hippocampal synaptic plasticity and cognition [55].

In the present study, a significant decline in the SIRT3 protein levels still existed 4 weeks after the rats were subjected to HIR, compared to those under sham operation. Similarly, the NOR results also proved that cognitive deficits were present in rats after HIR. This suggested that decreased levels of SIRT3 expression caused by brain damage during early life can persist to adulthood and disrupt cognitive function. However, treatment with Dex upregulated SIRT3 protein levels and activity, corroborating with previous research [36]. Treatment with Dex also led to an increase in the levels of recognition indexes. Moreover, treatment with the selective SIRT3 inhibitor, 3-TYP, reversed the positive effects of Dex. Intriguingly, Dex treatment had no such effects on the SIRT1 expression and activity, unlike previous studies in which Dex treatment played a protective role by activating SIRT1 pathway [56, 57]. Hence, we speculated that due to the limited ability of Dex treatment to increase the expression of SIRT1, its activation effect cannot be shown obviously when SIRT1 is already activated. The above data therefore suggests that the protective effects of Dex against HIR are partly mediated by the upregulation of SIRT3, which plays an important role in hippocampus damage and cognitive deficits.

Mitophagy is selective autophagy that degrades excess or damaged mitochondria through the lysosome system. This is an important mechanism for mitochondrial quality control as it ensures normal functioning of the organelle as well as homeostasis in cells and tissues [58]. Therefore, deficiencies in mitophagy result in the accumulation of dysfunctional mitochondria and oxidative stress. Recent evidence demonstrated that mitophagy played a protective role in brain injury [59, 60]. BINP3 is a marker of mitophagy, which interacts with LC3 then mediates the autophagic-lysosome degradation of damaged mitochondria [61]. Additionally, P62 is known to mediate autophagic degradation [62] and was recently regarded as an inducer of mitophagy. It facilitated the recruitment of damaged mitochondria to the phagophore by binding to LC3-II [63]. Moreover, FOXO3*α* was the first transcription factor, regulated by SIRT3 that was shown to promote autophagy [64]. Normally, FOXO3*α* directly controls the activation of mitophagy by facilitating the transcription of downstream autophagy-related genes, such as LC3 and BNIP3 [65]. Previous studies confirmed that Dex exerted protective effects by enhancing mitophagy [66]. Similarly, this study showed that treatment with Dex promoted mitophagy in the hippocampus evidenced by the increased expression levels of FOXO3*α*, BINP3, LC3-II/LC3-I, and P62. Moreover, TEM analysis demonstrated a marked increase in autophagy-like vesicles after Dex treatment. The slight rise in the expression levels of FOXO3*α*, BINP3, LC3-II/LC3-I, and P62 in the HIR group was noteworthy as this indicated that mitophagy was also activated after HIR.

These results suggested that mitophagy acted as a protective mechanism although it was unable to efficiently clean up damaged mitochondria in the hippocampus following a HIR challenge. Additionally, the slight increased levels of FOXO3*α* corresponded to the lowest value of SIRT3 in the HIR group, indicating that there were other signaling pathways involved in the regulation of mitophagy. However, 3-TYP treatment reversed the process of mitophagy induced by Dex. These results thus implied that Dex induced mitophagy in the hippocampus after HIR partly by activating the SIRT3/FOXO3*α*/BINP3 signaling pathway.

Microglial cells act as the resident macrophages in the central nervous system, and their activation represents the primary stage of neuroinflammation [67]. Moreover, activated microglia are responsible for the secretion of multiple neurotoxic agents such as cytokines, chemokines, and ROS that promote neuron apoptosis [68]. As an important deacetylase in mitochondria, SIRT3 modulates mitochondrial function and balances ROS homeostasis by deacetylating different downstream proteins. These include the antioxidative proteins SOD2 and PRDX3 which have been extensively investigated in oxidative stress. It is known that SOD2 converts superoxide into hydrogen peroxide (H_2_O_2_), which is further reduced by glutathione peroxidase in the mitochondria [69]. Recent research showed that PRDX3 scavenged almost 85-90% of H_2_O_2_. Therefore, it is the most predominant and efficient H_2_O_2_ detoxifying enzyme in the mitochondria [70]. Furthermore, previous studies showed that the upregulation of SIRT3-mediated SOD2 and PRDX3 deacetylation could enhance the mitochondrial antioxidant defense activity and alleviate oxidative damage to the mitochondria [71, 72]. Moreover, SIRT3 can promote SOD2 transcription by interacting with the FOXO3*α* proteins and activating the FOXO3*α*-dependent antioxidant-encoding gene SOD2 [73]. A similar regulation has been reported between SIRT1 and PRDX3 [74]. Considering that deacetylated SOD2 and PRDX3 exerted their antioxidant effects, we only determined the protein levels of SOD2 and PRDX3. In this study, both of SOD2 and PRDX3 were substantially decreased since HIR caused a significant decline in SIRT3. This resulted to oxidative stress as shown by the overactivation of microglia, accumulation of ROS, and MDA content as well as reduction in the activity of SOD.

Moreover, treatment with Dex upregulated both expression levels of SOD2 and PRDX3, eventually suppressing oxidative stress. However, the antioxidant effect of Dex was reversed by 3-TYP and 3-MA. Interestingly, expression levels of SOD2 and PRDX3 were not affected by 3-MA, indicating that mitophagy may be the last step in removing ROS and damaged mitochondria. Therefore, Dex inhibited activation of microglia and reduced oxidative stress in the hippocampus after HIR, mainly by enhancing SIRT3-mediated mitophagy.

Mitochondrial dysfunction is an indicator of various neurological disorders [75]. Moreover, oxidative stress can enhance the opening of the mitochondrial permeability transition pore (mPTP). This may lead to the collapse of the mPTP and a decrease in ATP production [76, 77] resulting to mitochondrial swelling and cell death [78]. It is well-known that mitophagy maintains mitochondrial quality and function by timely removal of damaged mitochondria. Additionally, CYP-D is well recognized as a key component of mPTP and plays an essential regulatory role in opening of the pore [79]. Previous research showed that hyperacetylation of CYP-D contributed to mPTP opening and subsequent cell death after myocardial ischemia-reperfusion. In addition, increased SIRT3 activity and subsequent attenuation of CYP-D acetylation prevented lethal injury after reperfusion [80]. Results from the present study were in line with those highlighted in previous literature.

In this study, HIR caused hippocampal mitochondrial dysfunction, evidenced by the decreased levels of MMP and ATP. Moreover, there was increased acetylation of CYP-D. Dex treatment effectively maintained hippocampal mitochondrial function after HIR partly by deacetylation of CYP-D, restored MMP, and increased ATP production. However, 3-TYP and 3-MA treatment reversed the beneficial effect of Dex. Notably, 3-MA did not affect the deacetylation of CYP-D. Based on these results, it was concluded that Dex activation of the SIRT3/SOD2/PRDX3 and SIRT3/CYP-D signaling pathways may be involved in antioxidative activity and improvement of mitochondrial function. However, SIRT3-mediated mitophagy played a dominant role in these processes by ensuring timely and effective removal of ROS and damaged mitochondria.

The inflammasome is a multiprotein complex of pattern-recognition receptors such as NLR families and absent in melanoma 2 (AIM2). Additionally, it consists of the bipartite adaptor protein referred to as the apoptosis speck-like protein containing ASC and the effector protein-pro-caspase-1 [81, 82]. Once the inflammasome is activated, pro-caspase-1 switches to active caspase-1, which converts IL-1*β* and IL-18 precursors into mature IL-1*β* and IL-18 [83, 84]. Moreover, active caspase-1 cleaves GSDMD, which forms the plasma membrane pores. This in turn induces cell swelling, rupture, and release of cytokines. The process is a proinflammatory form of programmed cell death termed as pyroptosis [85–87]. Accumulating evidence suggests that the NLRP3 inflammasome plays an important role in inflammation in the central nervous system [88, 89] and cognitive deficits [90]. Moreover, a previous study demonstrated that the NLRP3 inflammasome could be regulated by ROS and mitophagy in the reverse direction [13]. A separate study using a cerebral ischemia-reperfusion model also showed that Parkin-dependent mitophagy was required for the inhibition of the NLRP3 inflammasome [91].

Furthermore, SIRT3 was shown to counteract pyroptosis by promoting autophagy and inhibiting the generation of ROS [92]. In this study, treatment with Dex suppressed activation of the NLRP3 inflammasome evidenced by the low expression levels of NLRP3, cleaved-caspase-1, ASC, and GSDMD consistent with the NLRP3 immunohistochemical staining results. Moreover, there was significant inhibition of IL-1*β* and IL-18. Notably, treatment with 3-TYP and 3-MA partially reversed the inhibitory effect of Dex on the NLRP3 inflammasome. These results therefore suggested that Dex strongly activated SIRT3-mediated mitophagy. This in turn exerted potent antioxidant effects by decreasing the levels ROS, eventually inhibiting activation of the NLRP3 inflammasome.

In summary, the major findings of this study were as follows: (1) HIR could cause hippocampus injury and cognitive dysfunction. This was associated with a reduced level of SIRT3 in the hippocampus. (2) Dex protected the hippocampus and alleviated cognitive dysfunction induced by HIR by activating SIRT3. (3) Dex inhibited activation of the NLRP3 inflammasome caused by HIR mainly through SIRT3-mediated mitophagy.

Despite the insightful findings, this study had a few limitations. First, Dex was shown to protect against hepatic ischemia-reperfusion injury. Therefore, it cannot be ruled out that the neuroprotective effects of Dex were partly due to its role as a shield against hepatic injury. Second, due to lack of brain-specific SIRT3 knockout mice, it cannot be ascertained that Dex only activated the SIRT3 pathway in the brain. Third, the study only focused on SIRT3 expression without considering other SIRT families. Therefore, the contribution of other SIRT families such as SIRT1 was not taken into account. Fourth, despite showing that low expression of SIRT3 disrupted cognitive function, the specific mechanisms involved were not elucidated. This therefore needs to be explored in future.

## 5. Conclusion

In conclusion, this study demonstrated that Dex effectively attenuated HIR-induced hippocampus injury and improved cognitive function in young rats by activating SIRT3-mediated mitophagy and inhibiting activation of the NLRP3 inflammasome.

## Data Availability

Data supporting the findings of this study are included in the article.

## Supplementary Materials

Supplementary Materials Figures S1-S3: the effects of the 3-TYP, 3-MA, and vehicle treatment on brain injury. S1: histopathological structure of the hippocampal CA1 region (magnification ×100; scale bars 400 *μ*m). S2: the levels of serum brain damage markers: NSE and S100*β*. S3: representative images showing TUNEL staining in the hippocampal CA1. The number of apoptotic cells was detected by TUNEL (red pixels), and the nuclei were identified by DAPI staining (blue pixels) (magnification ×200; scale bars 50 *μ*m). Apoptosis index was calculated as follows: the number of apoptotic cells/the number of total cells × 100%. *n* = 5 per group. Data are presented as mean ± SEM.
