# Corrigendum to “Dexmedetomidine Ameliorates Hippocampus Injury and Cognitive Dysfunction Induced by Hepatic Ischemia/Reperfusion by Activating SIRT3-Mediated Mitophagy and Inhibiting Activation of the NLRP3 Inflammasome in Young Rats”

**DOI:** 10.1155/2021/9797165

**Published:** 2021-08-04

**Authors:** Wenli Yu, Jingshu Lyu, Lili Jia, Mingwei Sheng, Hongli Yu, Hongyin Du

**Affiliations:** ^1^Department of Anesthesiology, Tianjin First Center Hospital, Tianjin 300192, China; ^2^Tianjin Medical University First Center Clinical College, Tianjin 300070, China

In the article titled “Dexmedetomidine Ameliorates Hippocampus Injury and Cognitive Dysfunction Induced by Hepatic Ischemia/Reperfusion by Activating SIRT3-Mediated Mitophagy and Inhibiting Activation of the NLRP3 Inflammasome in Young Rats” [1], a number of errors have been identified, which should be corrected as follows:

1. In Section 2.2, paragraph 7, “(4) 3-TYP group where they were pretreated with 3-TYP then treated with 20 *μ*g/kg Dex before hepatic ischemia” should be corrected to, “(4) 3-TYP group where they were pretreated with 3-TYP then treated with 25 *μ*g/kg Dex before hepatic ischemia”.
2. The third limitation in paragraph 47 of the Discussion should not have appeared in the published article, “the study only focused on SIRT3 expression without considering other SIRT families. Therefore, the Oxidative Medicine and Cellular Longevity 13 contribution of other SIRT families such as SIRT1 was not taken into account.”
3. Due to an error introduced during the preparation of the manuscript, the IR DAPI panel of Figure 6 is duplicated with the S DAPI panel. The corrected figure is provided below
4. Due to an error introduced during the preparation of the manuscript, in Figure 3(d), SIRT3/GAPDH should be corrected to SIRT1/GAPDH. The corrected figure is provided below

## Figures and Tables

**Figure 1:**
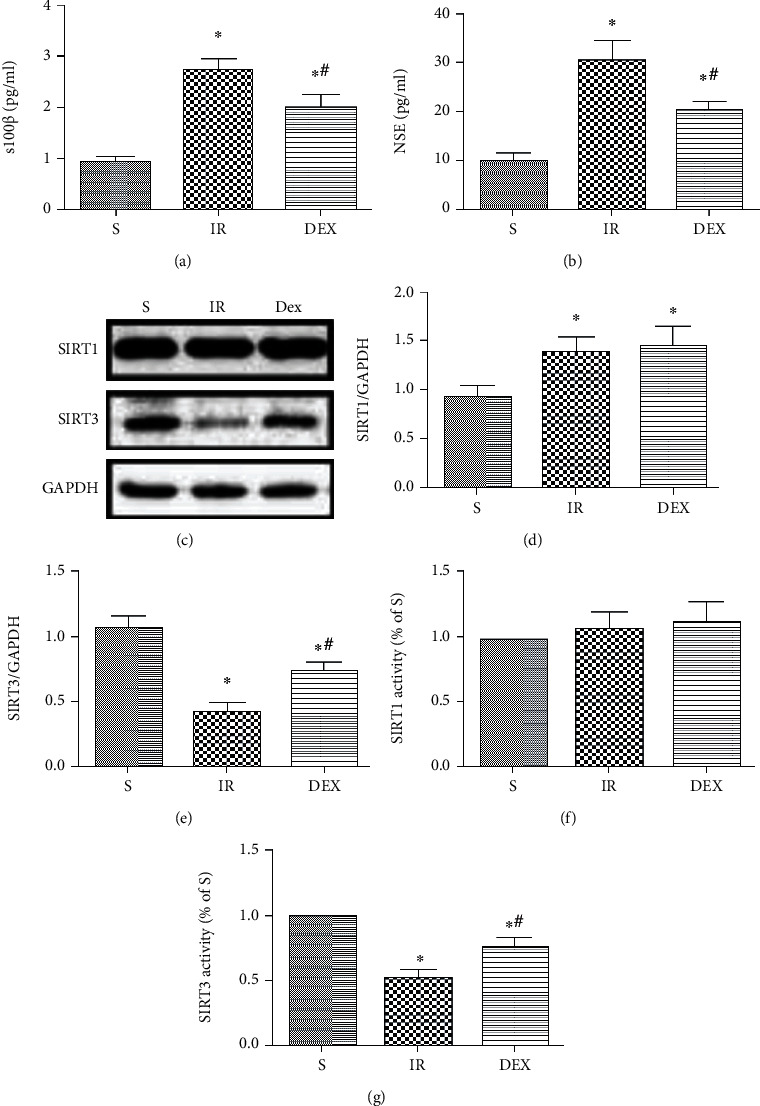
Dex decreased serum brain damage markers and upregulated SIRT3 expression and activity in the hippocampus after HIR. Serum levels of brain damage markers: (a) S100*β* and (b) NSE (b). (c) Expression of SIRT1 and SIRT3 in hippocampus tissues. (d) Quantitative analysis of expression level of SIRT1. (e) Quantitative analysis of expression level of SIRT3. (f) SIRT1 activity in mitochondria from hippocampus tissues. (g) SIRT3 activity in mitochondria from hippocampus tissues. *n* = 5 per group. Data are presented as mean ± SEM. ^∗^*P* < 0.05 vs. S group; ^#^*P* < 0.05 vs. IR group.

**Figure 2:**
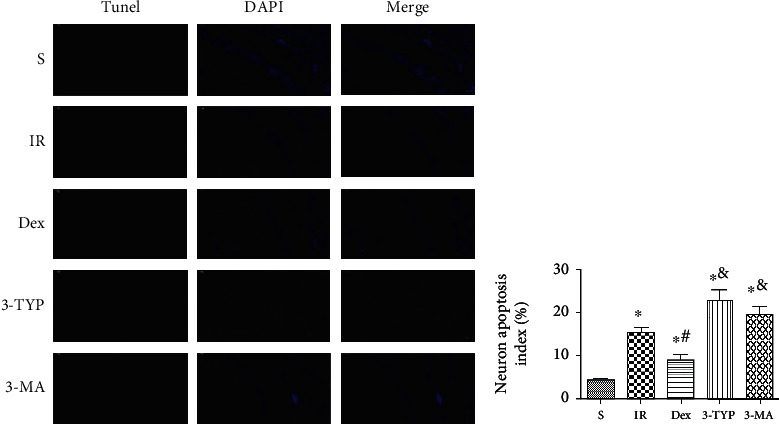
Dex attenuated neuronal apoptosis in hippocampus after HIR. Representative images show TUNEL staining in the hippocampal CA1. The number of apoptotic cells was detected by TUNEL (red pixels), and the nuclei were identified by DAPI staining (blue pixels) (magnification ×200; scale bars 50 *μ*m). Apoptosis index was calculated as follows: the number of apoptotic cells/the number of total cells × 100%.

## References

[1] Yu W., Lyu J., Jia L., Sheng M., Yu H., Du H. (2020). Dexmedetomidine ameliorates hippocampus injury and cognitive dysfunction induced by hepatic ischemia/reperfusion by activating SIRT3-mediated mitophagy and inhibiting activation of the NLRP3 inflammasome in young rats. *Oxidative Medicine and Cellular Longevity*.

