# Supplementary material for: Dexmedetomidine Ameliorates Hippocampus Injury and Cognitive Dysfunction Induced by Hepatic Ischemia/Reperfusion by Activating SIRT3-Mediated Mitophagy and Inhibiting Activation of the NLRP3 Inflammasome in Young Rats
Source: Oxid Med Cell Longev. 2020 Nov 20;2020:7385458. doi: 10.1155/2020/7385458 (PMC8418694; doi:10.1155/2020/7385458)
Supplement: Supplementary Materials — Figures S1-S3: the effects of the 3-TYP, 3-MA, and vehicle treatment on brain injury. S1: histopathological structure of the hippocampal CA1 region (magnification ×100; scale bars 400 μm). S2: the levels of serum brain damage markers: NSE and S100β. S3: representative images showing TUNEL staining in the hippocampal CA1. The number of apoptotic cells was detected by TUNEL (red pixels), and the nuclei were identified by DAPI staining (blue pixels) (magnification ×200; scale bars 50 μm). Apoptosis index was calculated as follows: the number of apoptotic cells/the number of total cells × 100%. n = 5 per group. Data are presented as mean ± SEM. [file 7385458.f1.docx]

Supplementary Information


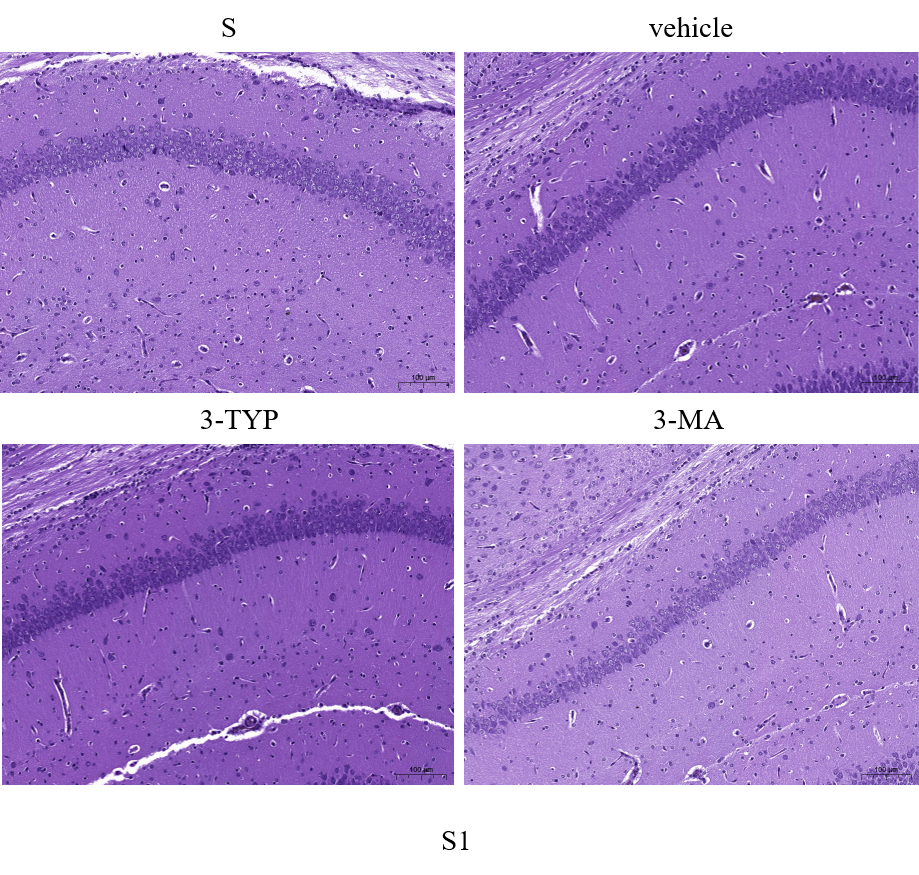


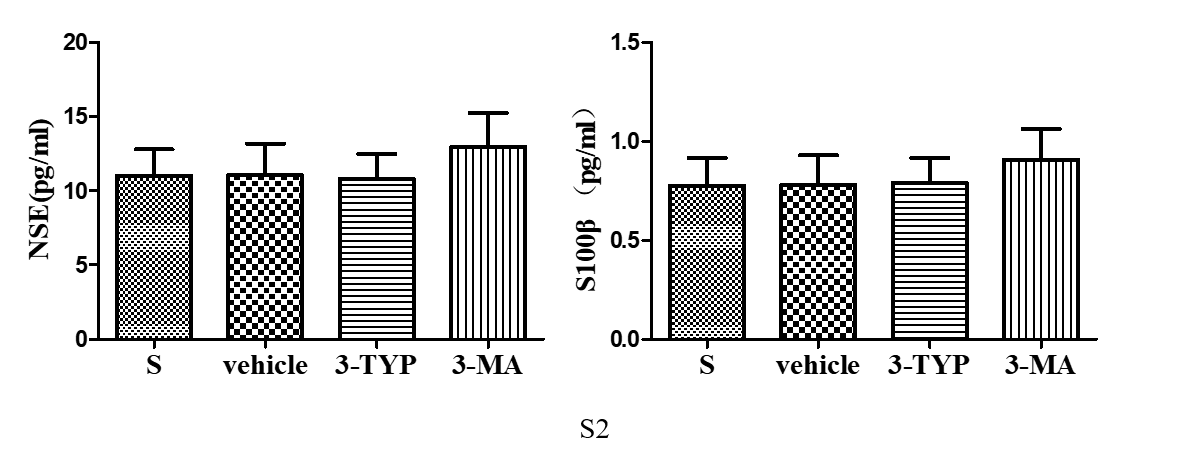


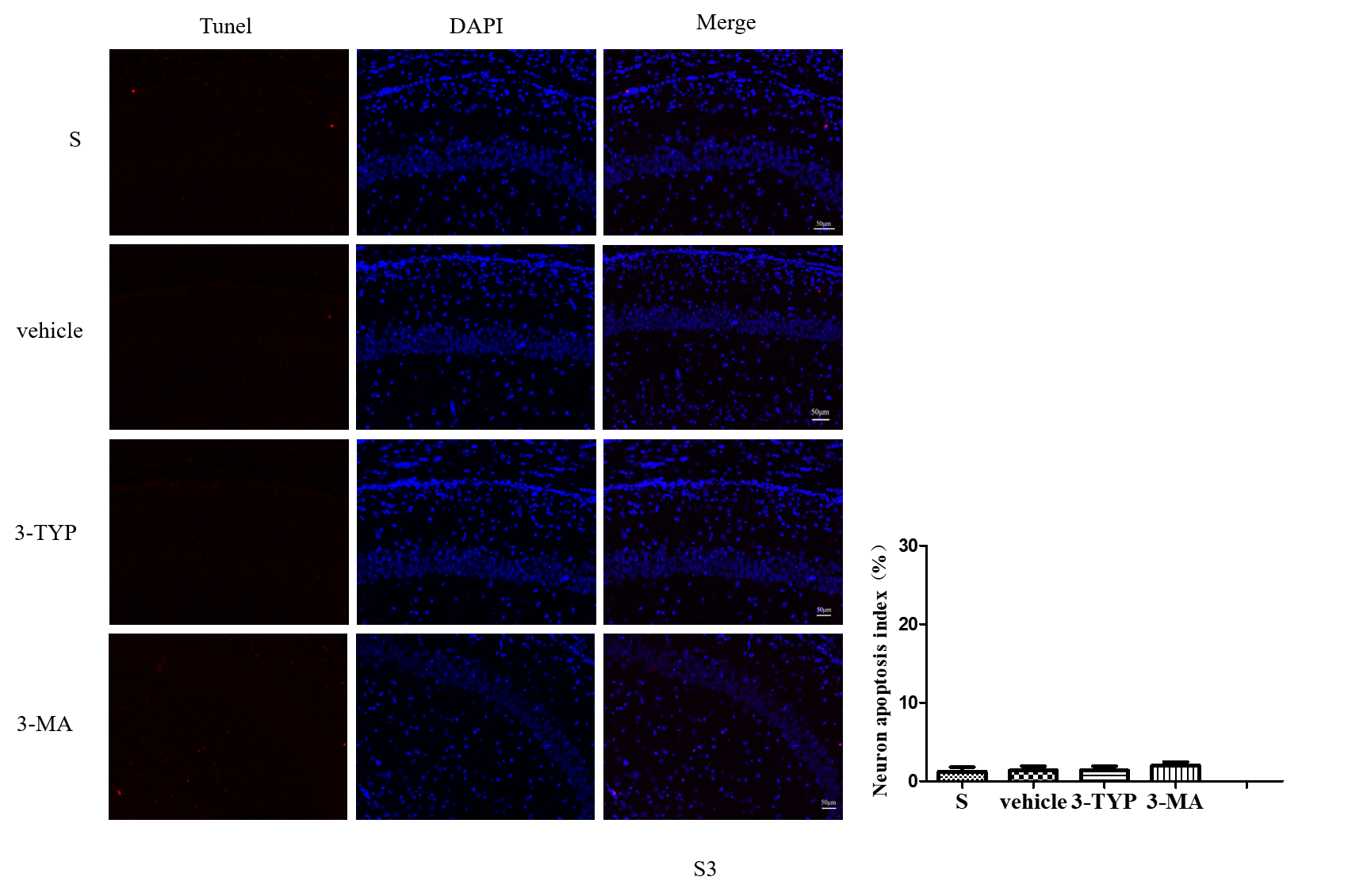


FIGURE S1-S3. The effects of the 3-TYP, 3-MA and vehicle treatment on brain injury. (S1) Histopathological structure of the hippocampal CA1 region (magnification × 100, scale bars 400µm).

(S2) The levels of serum brain damage markers: NSE andS100β. (S3) Representative images showing TUNEL staining in the hippocampal CA1. The number of apoptotic cells was detected by TUNEL (red pixels), and the nuclei were identified by DAPI staining (blue pixels) (magnification × 200, scale bars 50 um). Apoptosis index was calculated as: (the number of apoptotic cells / the number of total cells × 100%). n = 5 per group. Data are presented as mean±SEM.
